# Supplementary material for: Control of Silver Coating on Raman Label Incorporated Gold Nanoparticles Assembled Silica Nanoparticles
Source: Int J Mol Sci. 2019 Mar 13;20(6):1258. doi: 10.3390/ijms20061258 (PMC6471565; doi:10.3390/ijms20061258)
Supplement: Supplementary file 1 [file ijms-20-01258-s001.pdf]

Control of Silver Coating on a Raman Labeling Chemical Incorporated Gold Nanoparticles Assembled Silica Nanoparticle

Supplementary Materials:

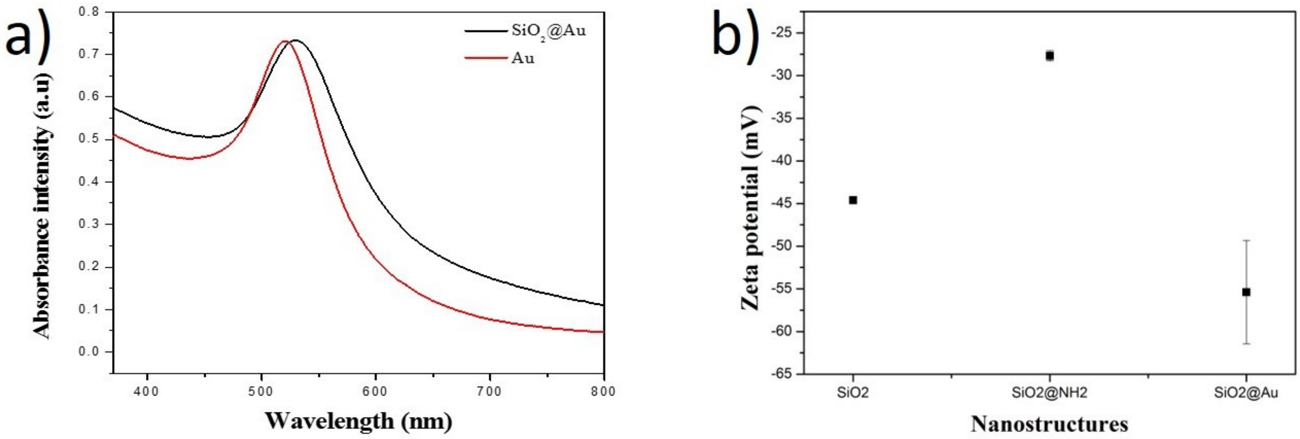

Figure S1. (a) UV-Vis of Au and SiO_2@Au and (b) zeta potential of silica nanoparticles (SiO_2), 3-aminopropyltriethoxysilane (APTS) coated silica nanoparticles (SiO_2@NH_2) and gold embedded silica nanoparticles (SiO_2@Au).

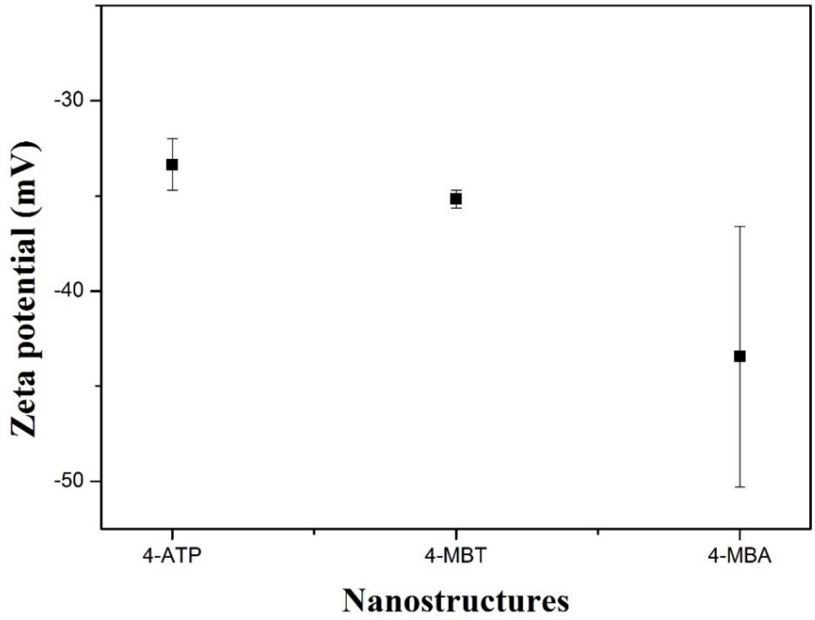

Figure S2. Zeta potential of SiO_2@Au, SiO_2@Au@4-ATP, SiO_2@Au@4-MBT and SiO_2@Au@4-MBA.

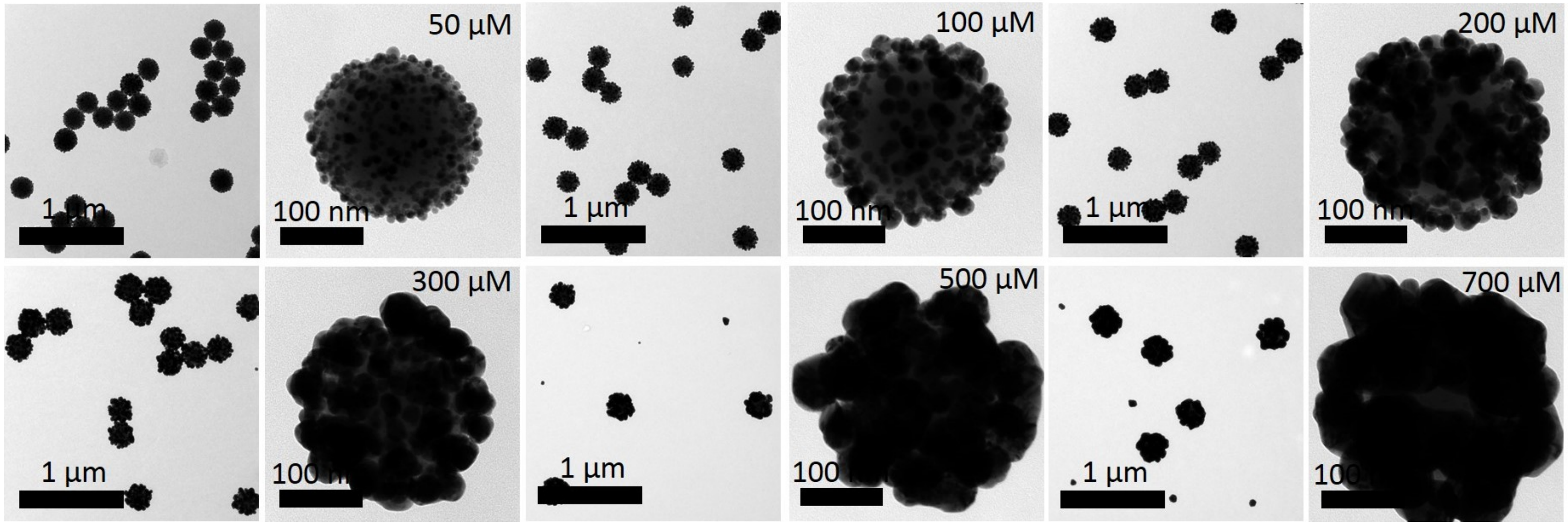

Figure S3. Transmission electron microscopy (TEM) images of SiO_2@Au-4-MBA@Ag nanoparticles coated with different concentrations of $AgNO_3$ in water. Concentration of 4-MBA was 1 mM.

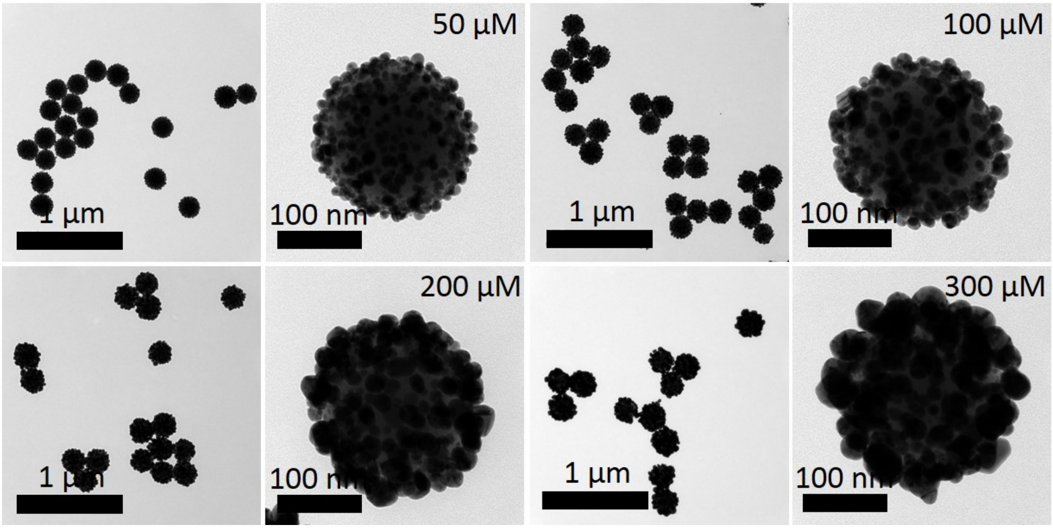

Figure S4. TEM images of SiO_2@Au@4-ATP@Ag nanoparticles coated with different concentrations of $AgNO_3$. Concentration of 4-ATP was 1 mM.

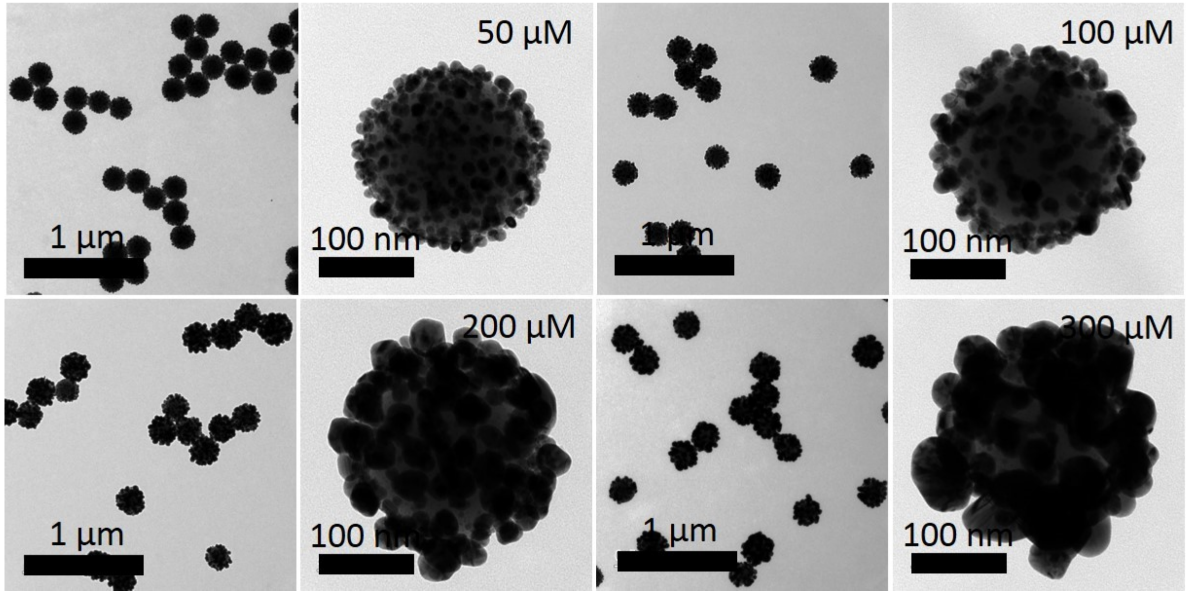

Figure S5. TEM images of SiO_2@Au@4-MBT@Ag nanoparticles coated with different concentrations of $AgNO_3$. Concentration of 4-MBT was 1 mM.

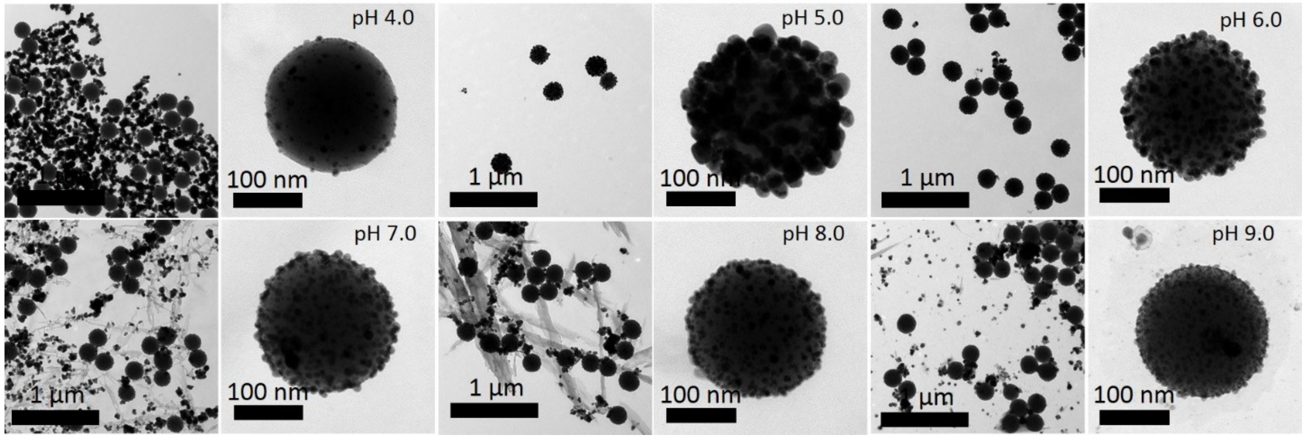

Figure S6. TEM images of SiO_2@Au@4-MBT@Ag nanoparticles coated at different pH. Concentration of 4-MBT was 1 mM.

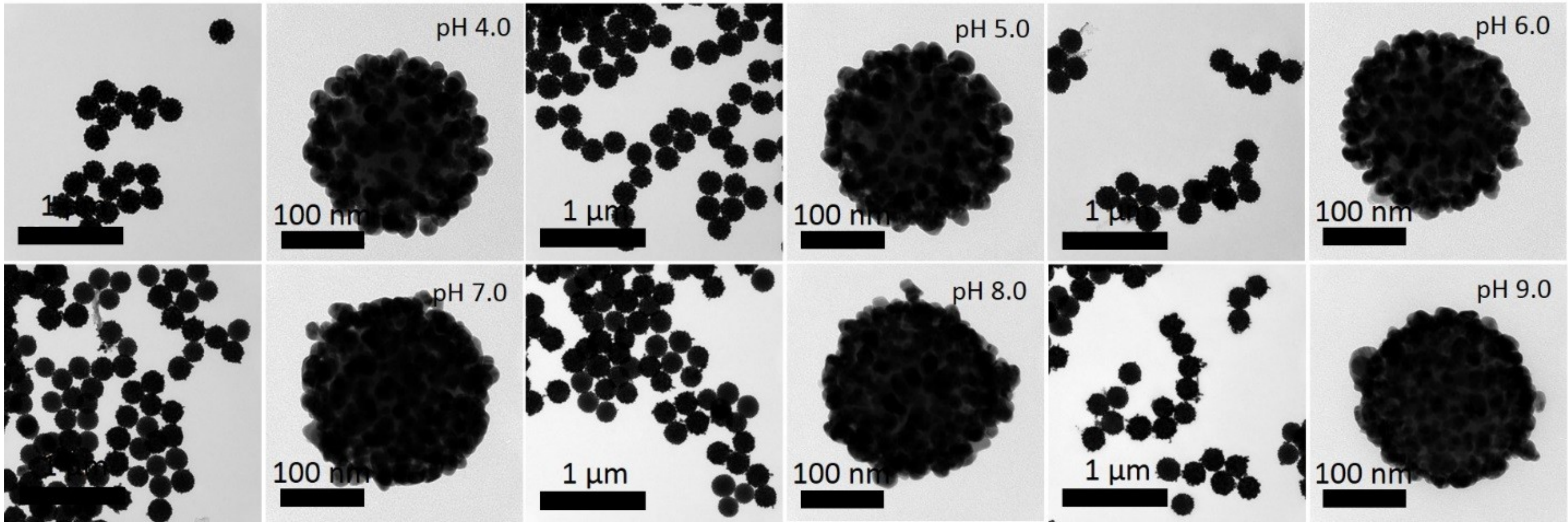

Figure S7. TEM images of SiO_2@Au@4-ATP@Ag nanoparticles coated at different pH. Concentration of 4-ATP was 1 mM.

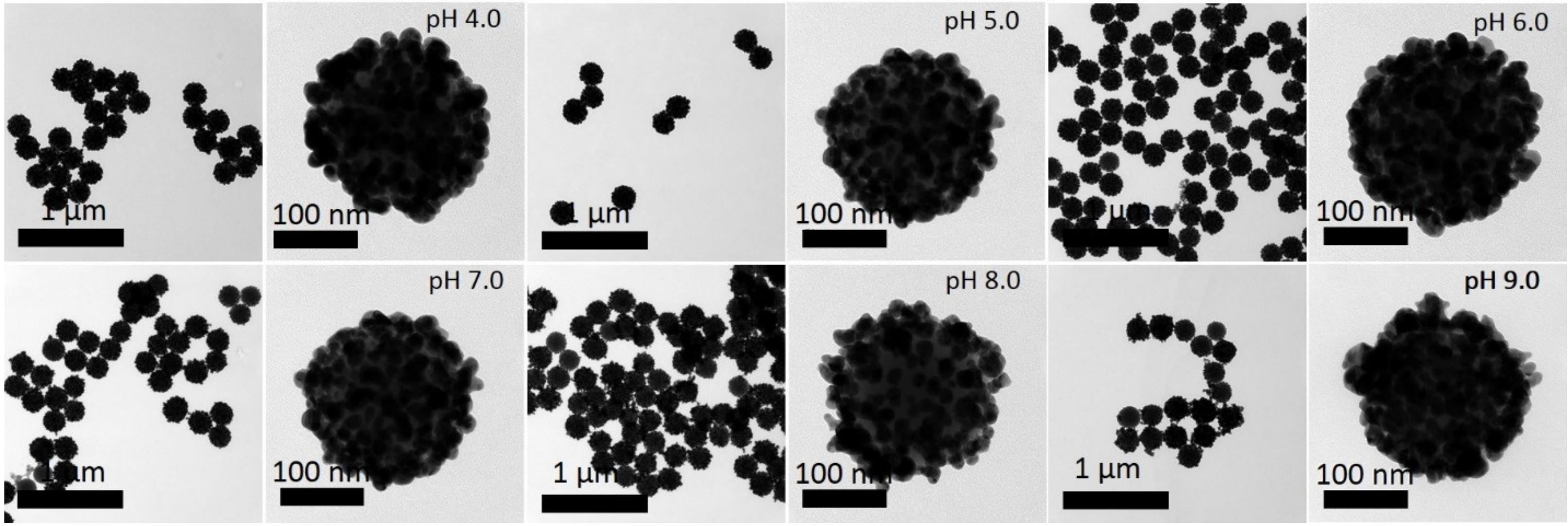

Figure S8. TEM images of SiO_2@Au@4-MBA@Ag nanoparticles coated at different pH. Concentration of 4-MBA was 1 mM.
